# Exendin-4 Caused Growth Arrest by Regulating Sugar Metabolism in *Hyphantria cunea* (Lepidoptera: Erebidae) Larvae

**DOI:** 10.3390/insects15070503

**Published:** 2024-07-05

**Authors:** Wenhui Shi, Lu Zhang, Yuecheng Zhao, Xingpeng Li

**Affiliations:** 1 College of Wildlife and Protected Area, Northeast Forestry University, Harbin 150040, China; 13115512001@163.com; 2 College of Forestry, Northeast Forestry University, Harbin 150040, China; zhangl@nefu.edu.cn; 3 College of Forestry and Grassland Science, Jilin Agricultural University, Changchun 130118, China; 4 College of Forestry, Beihua University, Jilin 132013, China

**Keywords:** exendin-4, *Hyphantria cunea*, nutritional indices, glycolysis, AMPK, insulin-like peptides

## Abstract

**Simple Summary:**

This study investigated the influence of exendin-4, a glucagon-like peptide-1 receptor (GLP-1R) agonist, on fall webworm *Hyphantria cunea* larvae. We found that exendin-4 induced growth arrest by regulating sugar metabolism, affecting digestive enzyme activities, and altering metabolite profiles. Specifically, it decreased glucose and trehalose levels while increasing the glycogen content, indicating a regulatory effect on blood sugar. Exendin-4 also promoted glycolysis and reduced ATP levels, potentially through the activation of AMPK. These findings enhance our understanding of the physiological effects of GLP-1R agonists in insects and could inform pest management strategies.

**Abstract:**

Insects’ growth and development are highly dependent on energy supply, with sugar metabolism playing a pivotal role in maintaining homeostasis and regulating physiological processes. The present study investigated the effects of exendin-4, a glucagon-like peptide-1 receptor (GLP-1R) agonist, on the growth, development, glycolysis, and energy metabolism of fourth-instar larvae of the fall webworm, *Hyphantria cunea*. We determined the impact of exendin-4 on larval growth and nutritional indices, analyzed the responses of glycolytic and metabolic pathways, and revealed the underlying regulatory mechanisms. Exendin-4 treatment significantly decreased growth and nutritional indices, influenced the activity of digestive enzymes, and induced changes in metabolite profiles, particularly affecting energy substance metabolism. We observed an increase in the glycogen content and a decrease in glucose and trehalose levels in the hemolymph, suggesting a regulatory effect on blood sugar homeostasis. Furthermore, exendin-4 promoted glycolysis by enhancing the activities and expressions of key glycolytic enzymes, leading to an increase in pyruvate production. This was accompanied by a reduction in ATP levels and the activation of AMP-activated protein kinase (AMPK), which may underlie the growth arrest in larvae. Our findings provide novel insights into the effects of exendin-4 on insect responses from an energy metabolism perspective and may contribute to the development of GLP-1R agonists for pest management.

## 1. Introduction

Insects’ growth and development heavily rely on energy supply. They primarily acquire energy substances through feeding, with nutrients being absorbed in the midgut and converted into more readily storable and transportable forms, such as trehalose and glycogen, within the fat body [1,2]. The transformation of energy substances and energy metabolism in insects are regulated by hormones and various signaling pathways, enabling insects to adapt to environmental changes and optimize their growth and survival strategies in different physiological states [3,4,5]. Among these, glucose metabolism plays a crucial role in maintaining sugar homeostasis in hemolymph, regulating cuticle chitin synthesis, and other physiological processes, thus garnering widespread attention and research [6].

Insects mainly participate in carbohydrate metabolism in the form of glucose and trehalose [7,8]. Trehalose, which serves as the primary sugar substance in hemolymph, is commonly known as “insect blood sugar” [9]. The regulatory system of insect hemolymph sugar exhibits evolutionary conservatism with the mammalian pancreatic islet endocrine system [10]. In mammals, blood sugar is regulated by pancreatic α- and β-cells that secrete glucagon and insulin. Despite the absence of a complete pancreatic islet tissue, studies on model insects such as *Bombyx mori* and *Drosophila melanogaster* have confirmed that they produce insulin-like peptides (ILPs) via insulin-secreting cells and regulate blood sugar homeostasis via antagonistic regulation by the adipokinetic hormone (AKH) secreted by the corpora cardiaca [11,12,13]. ILP gene-deficient *Drosophila* larvae exhibit poor development and elevated circulating carbohydrate levels, similar to symptoms of type I diabetes; conversely, disruption of AKH cell secretion leads to decreased circulating carbohydrate levels and significantly reduces the trehalose content in the hemolymph [14,15]. This indicates that insects possess a complete molecular mechanism for sensing and regulating blood sugar levels. Moreover, insects utilize the insulin and insulin-like growth factor signaling pathway (IIS) to mediate blood sugar homeostasis and growth development in larvae, as well as reproductive capacity and lifespan in adults [16,17,18]. Therefore, in-depth research into the regulation of carbohydrates contributes to unveiling the physiological responses and metabolic functions in insects.

Glycolysis plays an irreplaceable role in regulating blood sugar and glycogen levels in insects, thereby affecting molting, pupation, and eclosion by changes in glycolytic flux [19,20]. In animal cells, hexokinase (HK), 6-phosphofructokinase (PFK), and pyruvate kinase (PK) are rate-limiting enzymes mediating the only three irreversible reactions in glycolysis [21,22]. HK, PFK, and PK are allosterically regulated by multiple factors and signaling pathways, including the ADP/ATP ratio, insulin/glucagon, the forkhead box O (FoxO) signaling pathway, AMP-activated protein kinase (AMPK), and the mammalian target of rapamycin (mTOR) and phosphatidylinositol 3-kinase/protein kinase B (P13K/Akt) signaling pathway. In particular, PFK and PK are regulated via allosteric inhibition by ATP and covalent modifications by insulin [23,24,25,26]. Pyruvate (PA), the final product of glycolysis, influences the ATP levels in insects through the tricarboxylic acid (TCA) cycle and subsequently impacts the growth, absorption, and conversion of nutrients at both the phenotypic and physiological levels [27,28,29]. Previous research has shown that insects possess glycolytic regulation mechanisms similar to those in higher animals at the cellular level [30,31]. However, given their unique physiological structures and metabolic adaptations, insects require further research into their responses to regulators of sugar metabolism and glycolysis.

As an important hormone regulating mammalian blood sugar homeostasis, glucagon-like peptide-1 (GLP-1) is secreted by L-cells in the jejunum and colon and acts on GLP-1 receptors (GLP-1Rs) [32]. GLP-1R agonists, such as exendin-4 (Figure S1), mimic the natural incretin hormone GLP-1 by binding to its receptor, which activates the cAMP/PKA pathway [33,34]. This activation promotes calcium influx through L-type voltage-gated Ca^2+^ channels and triggers calcium release from the endoplasmic reticulum, thereby activating calmodulin and significantly enhancing insulin exocytosis [35,36,37]. In addition to promoting the synthesis and secretion of insulin, GLP-1R agonists also slow gastric emptying and reduce food intake, thereby helping to reduce body weight in patients [38]. Interestingly, insects possess receptors similar to the GLP-1Rs found in vertebrates. Unlike their vertebrate counterparts, insect GLP-1Rs do not directly regulate blood sugar levels but interact with neuropeptides that modulate energy metabolism, particularly during periods of high energy demand [39,40]. For instance, adipokinetic hormone (AKH) serves as the insect equivalent of a glucagon-like peptide [41]. AKH’s mechanism of action includes signaling through G-protein-coupled receptors, leading to the production of energy-providing substrates such as trehalose, diacylglycerol, or proline, thus mobilizing energy reserves in a manner analogous to glucagon’s role in glucose homeostasis in mammals [42]. Therefore, due to potential structural and functional differences between insect and mammalian GLP-1 receptors, the regulatory effects of GLP-1R agonists on insects may also differ.

According to previous research, several medications prescribed for treating type II diabetes have shown a capacity to impact insect growth and carbohydrate metabolism. For example, metformin significantly affects carbohydrate levels and delays molting in *Hyphantria cunea* larvae through the activation of AMPK [43]. The sulfonylurea (SU) compound glibenclamide interacts with SU receptors on insect epidermal cells to inhibit K_ATP_ channels, thereby disrupting chitin biosynthesis through the blockage of N-acetylglucosamine transmembrane transport [44,45]. Additionally, benzoylphenylureas, which are chemically similar to SUs, arrest the growth of *H. cunea* larvae and activate glycolysis [46,47]. Although GLP-1R agonists represent an emerging class of antidiabetic drugs, the mechanisms by which they affect insect physiology remain poorly understood, especially energy metabolism.

The fall webworm (FWW), *Hyphantria cunea* (Lepidoptera: Erebidae), is a highly destructive and polyphagous forest pest known to colonize over 600 hosts [48]. In the present study, we investigated the effects of exendin-4, a representative GLP-1R agonist, on growth and development, glycolysis, and energy metabolism in *H. cunea* fourth-instar larvae. The objectives were to (1) determine the changes in the growth and nutritional indices of *H. cunea* in response to exendin-4; (2) analyze the responses of related glycolytic and metabolic pathways to exendin-4; and (3) reveal the regulatory mechanism of exendin-4 in *H. cunea* larvae. The findings provide novel insights into the effects of exendin-4 on insect responses from the perspective of energy metabolism and may lay the groundwork for a potential application of GLP-1R agonists for pest management.

## 2. Materials and Methods

### 2.1. Insect Rearing and Exendin-4 Treatment

Eggs of *H. cunea* and artificial diets were acquired from the Chinese Academy of Forestry (Beijing, China). The newly hatched larvae were reared in an incubator maintained at a temperature of 25 ± 1 °C and a relative humidity of 65% ± 5%, with a 16 h light–8 h dark (16 L–8 D) photoperiod. The hatching larvae were fed artificial diets, and newly molted fourth-instar larvae were selected and starved for 24 h for subsequent experiments.

Exendin-4 was purchased from MedChemExpress (Monmouth Junction, NJ, USA). Since no effects of PBS on various substances and glycolytic pathways were observed, we did not run the analysis of normal controls (larvae without treatment) (Figure S2). Five concentrations of exendin-4 (0, 0.1, 1, 10, and 100 ppm) were dissolved in 0.01 mM phosphate-buffered saline (PBS) (pH 7.0), and 0.5 μL was injected into the larvae using a microinjector (HAMILTON, Reno, NV, USA). The experiment was organized into five groups (n = 30 in each group), with three biological replicates. The virulence equation was calculated by counting the number of dead individuals over a period of 72 h, and the LC_20_ (20%-lethal concentration) and LC_50_ (half-lethal concentration) were determined.

Starvation-treated larvae were divided into three groups (n = 50 in each group) and treated with LC_20_ and LC_50_ concentrations of exendin-4 in 0.01 mM PBS (pH 7.0) or 0.01 mM PBS (pH 7.0) as a control (CK), with three biological replicates. Then, the larvae weight, diet dry weight, and feces dry weight were recorded at 0, 24, 48, and 72 h after the artificial diet feeding. The nutritional indices were calculated using the following equations:

Efficiency of conversation of ingested food, ECI (%) = G/I × 100;

Efficiency of conversation of digested food, ECD (%) = G/I − F) × 100;

Relative consumption rate, RCR (g/g·d) = I/(B × T);

Relative growth rate, RGR (g/g·d) = G/(B × T);

Approximate digestibility, AD (%) =(I − F)/I × 100.

where G = larval weight after feeding − larval weight before feeding; B = (larval weight after feeding + larval weight before feeding)/2; I represents the difference in the diet dry weights before and after larvae feeding; F represents the feces dry weight; and T is the duration of the experiment in days [47,49]. Based on the results of the nutritional indices, LC_20_ was chosen for the following measurements.

### 2.2. LC-MS-Based Metabolomics Analysis

#### 2.2.1. Metabolite Extraction

The 4th-instar *H. cunea* larvae were treated with LC_20_ exendin-4 (T) and PBS (CK) as described above, with six biological replicates for each group (n = 15). Metabolite extraction was performed following the methods described by Dunn et al. [50]. Briefly, the fat body tissues of larvae were dissected on ice. Then, the samples were ground into powders using liquid nitrogen, and 25 mg of each sample was weighed. An extract solution containing acetonitrile, methanol, and water in a 2:2:1 ratio, along with 2 μg/mL of L-2-chlorophenylalanine, was added. After vortexing for 30 s, the samples were homogenized at 35 Hz for 4 min and sonicated for 5 min in ice, which was repeated twice. The samples were then incubated at −40 °C for 1 h and centrifuged at 10,000 rpm for 15 min at 4 °C. A 600 μL sample of each supernatant was transferred and dried in a vacuum concentrator at 37 °C, ensuring the optimal sample concentration for subsequent LC/MS analysis. The dried samples were reconstituted in 200 μL of 50% acetonitrile by sonication on ice for 10 min. The samples were then centrifuged at 12,500 rpm for 15 min at 4 °C, and 60 μL of each supernatant was transferred for LC/MS analysis. A quality control (QC) sample was created by mixing an equal volume (10 μL) from all samples.

#### 2.2.2. LC-MS/MS Analysis

The UHPLC separation was performed using an Agilent 1290 UHPLC System (Agilent Technologies, Santa Clara, CA, USA) and a UPLC BEH Amide column, following established protocols [51,52]. The column and auto-sampler temperatures were maintained at 25 °C and 4 °C, respectively. The mobile phase comprised 25 mmol/L ammonium acetate and 25 mmol/L ammonia hydroxide in water (pH = 9.75) (A) and acetonitrile (B). The elution gradient was as follows: 0~0.5 min, 95% B; 0.5~7.0 min, 95%~65% B; 7.0~8.0 min, 65%~40% B; 8.0~9.0 min, 40% B; 9.0~9.1 min, 40%~95% B; and 9.1~12.0 min, 95% B. The injection volume for both positive and negative modes was 2 μL. The eluted metabolites were analyzed using a TripleTOF 6600 mass spectrometry (Sciex, Framingham, MA, USA) system, with data evaluation performed by the Analyst TF 1.7 software [50]. The MS/MS analysis targeted the 12 most intense precursor ions per cycle (0.56 s), with the ESI source conditions set at 60 psi for gas 1, gas 2, and the curtain gas. The source temperature was maintained at 600 °C, with a declustering potential of 60 V and the ion spray floating voltage at 5000 V (positive mode) or −4000 V (negative mode). 

#### 2.2.3. Data Analysis

Metabolite features detected in <20% of experimental samples or <50% of QC samples were excluded from the data analysis. Missing values in the raw data were imputed by half of the minimum value. Then, features with a relative standard deviation (RSD) greater than 30% were removed. The resulting three-dimensional data were subjected to analysis using the R package metaX (version 4.1.1) for the principal component analysis (PCA) [53]. Metabolites with variable importance in projection (VIP) values above 1.0 were initially identified as significantly altered, and the remaining variables were further evaluated using a Student’s *t*-test (Q-value > 0.05). Additionally, metabolite pathways were elucidated using the KEGG (http://www.kegg.jp, accessed on 1 July 2024) and MetaboAnalyst (http://www.metaboanalyst.ca/, accessed on 1 July 2024) databases.

### 2.3. Enzyme Activity Assay

For enzyme activity analysis, the *H. cunea* larvae were treated with the LC_20_ of exendin-4 (T) and PBS (CK), with three biological replicates per group (n = 20). To determine the activities of digestive enzymes, larvae midgut tissues were collected and dissected at 0 h, 24 h, 48 h, and 72 h, followed by rapid freezing in liquid nitrogen and subsequent grinding. The activities of α-amylase, lipase, and trypsin were determined using assay kits (Solarbio Co., Ltd., Beijing, China). The details for the sample treatment were conducted according to the manufacturer’s instructions. The principles of the assay kits were based on the rates of hydrolysis catalyzed by the three enzymes in the samples. The enzyme activities of α-amylase, lipase, and trypsin were measured using a Multiskan FC microplate reader (Thermo Fisher Scientific, Waltham, MA, USA) at wavelengths of 540 nm, 710 nm, and 253 nm, respectively. The enzyme activities in the samples were calculated based on the absorbance readings.

The extraction of soluble and membrane-bound trehalase from larvae was performed following previously described methods [54,55]. The fat body tissues of larvae were dissected on ice. Then, the samples were flash-frozen in liquid nitrogen and stored at −80 °C until further use. An amount of 20 mg of each sample was homogenized in 200 μL of 20 mM PBS (pH 6.0) on ice, followed by centrifugation at 15,100 *g* for 15 min at 4 °C. The resulting supernatant was collected as the soluble trehalase solution, while the precipitate was resuspended in 200 μL of 20 mM PBS (pH 6.0) and subjected to centrifugation at 15,100 *g* for 15 min at 4 °C to obtain the crude membrane-bound trehalase solution. The trehalase activity was determined using the trehalase assay kit (Nanjing Jiancheng Bioengineering Institute, Nanjing, China), which was quantified by measuring the amount of glucose produced from trehalose catalyzed by trehalase, expressed as μmol glucose/g tissue/min.

The activities of key enzymes involved in glycolysis, namely, hexokinase (HK), 6-phosphofructokinase (PFK), and pyruvate kinase (PK), were measured. The fat body samples were homogenized in 0.9 mL of a 0.9% NaCl solution, followed by centrifugation at 3000 rpm for 15 min at 4 °C. The resulting supernatants were collected for analysis. The activities of HK, PF, and PK were determined using assay kits obtained from Sangon Biotech (Shanghai, China), following the manufacturer’s protocols. Spectrophotometric measurements were performed with a Multiskan FC microplate reader (Thermo Fisher Scientific) at OD 340 nm. The data from each sample were recorded, and the enzyme activities were calculated accordingly.

### 2.4. Measurements of Carbohydrates, PA, and ATP

To determine the contents of trehalose and glucose, the larvae were punctured in the abdomen by a microinjector (HAMILTON, Reno, NV, USA). An amount of 2 μ of hemolymph was extracted from each larva, with three biological replicates for both the T and CK groups (n = 20). Then, the collected hemolymph was diluted with acetonitrile to fourfold in each group. The hemolymph was added to phenylthiourea (1–5% of the total volume) and stored at 4 °C. Subsequently, the content of larval blood sugar was analyzed using high-performance liquid chromatography (HPLC) with a refractive index detector (RID 2410) [56]. An 80:20 mixture of acetonitrile and water served as the mobile phase in a carbohydrate column (8 mm × 300 mL, Waters), with a flow rate of 0.6 mL/min and a detector temperature of 37 °C. All analyses were conducted in triplicate. Standard solutions of trehalose and glucose (Dr. Ehrenstorfer, Augsburg, Germany) were also produced in the same column to establish the standard curves. Finally, the levels of trehalose and glucose in the samples were determined using the generated standard curves.

To measure the levels of glycogen, adenosine triphosphate (ATP), and pyruvic acid (PA) in larvae, assay methods using corresponding kits (Nanjing Jiancheng Bioengineering Institute, Nanjing, China) were utilized, with three biological replicates for each group (n = 20). For the glycogen assay, the supernatant from the fat body was processed as in Section 2.3. Following the kit instructions, the dehydration of glycogen under strongly acidic conditions (H_2_SO_4_) and subsequent reaction with anthrone were measured at 620 nm. The glycogen contents in the samples were then calculated. Regarding the ATP and PA assays, the fat body samples were homogenized at 0 °C using an extractive agent (0.1 g of tissue–1 g of the agent) for 30 min. The homogenates were then centrifuged at 8000 rpm, and the supernatants were collected for analysis following the kit instructions. The protein content in each sample was measured using the Bradford method, and ATP and PA levels were defined as the amount per gram of protein.

### 2.5. Quantitative RT-PCR (qRT-PCR)

To extract total RNA from the larval fat body, the method for obtaining fat body samples followed the steps in Section 2.3 above. Then total RNA was extracted using TRIzol reagent (Tiangen Biotech, Beijing, China) according to the manufacturer’s instructions. RNA integrity was assessed using gel electrophoresis, while the purity and concentration of RNA were determined by measuring the OD_260_ and OD_280_ using a Multiskan FC microplate reader (Thermo Fisher Scientific). Subsequently, 0.5 μg of total RNA from each sample was used to synthesize cDNA templates using the gDNA Dispelling RT SuperMix kit (Tiangen Biotech, Beijing, China). The synthesized cDNAs were diluted 10-fold in sterile water and used as templates using the PreMix Plus (SYBR Green) fluorescent dye (Tiangen Biotech, Beijing, China) for qRT-PCR. In each reaction, the 20 μL final volume comprised 2 μL of the cDNA templates, 0.8 μL of each primer (10 μM), 10 μL of SuperReal PreMix Plus, and 6.4 μL of RNase-free ddH_2_O. The conditions for the qRT-PCR were set as follows: an initial denaturation at 95 °C for 15 min, followed by 40 cycles at 95 °C for 5 s, 55 °C for 30 s, and 72 °C for 32 s. A final melting curve analysis was conducted to confirm the amplification. The relative expression of mRNA was normalized to a reference gene (*Hcβ-actin*) and calculated using the 2^−ΔΔCt^ method. Table S1 provides the GenBank numbers and primer sequences used for the genes, along with the reference gene. Each sample was analyzed in triplicate with three biological replicates.

### 2.6. Statistical Analysis

The data for the experiments were recorded and organized using Excel 2016 and presented as means ± standard error (SEM). Statistical analysis was performed using SPSS 22.0 for Windows, including variance analysis and linear regression analysis. The mean values of two continuous normally distributed variables were compared by an independent-samples Student’s test (*p* < 0.05). Tukey’s test compared the statistical significance between the T and CK groups, with asterisks indicating significant differences (* *p* < 0.05, ** *p* < 0.01, and *** *p* < 0.001).

## 3. Results

### 3.1. The Toxicity Test of Exendin-4

**Figure 1:**
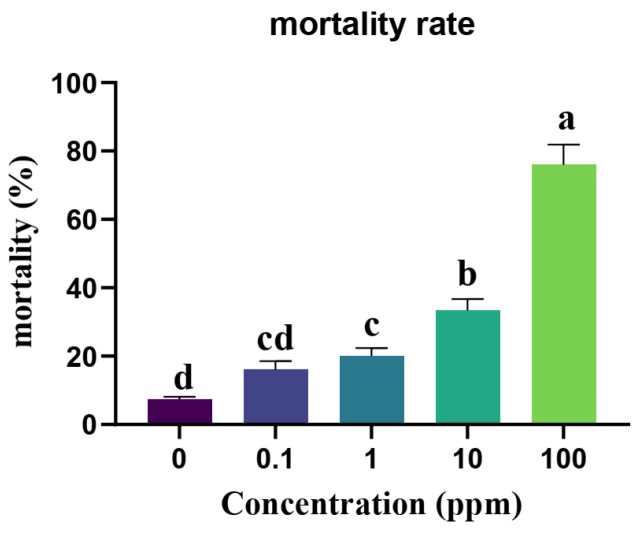
Toxicity test of exendin-4. Dose–mortality response relationship in *H. cunea* larvae after injected with 0 (PBS), 0.1, 1, 10, and 100 ppm of exendin-4 at 72 h. Data are presented as the means ± SE of three independent biological replications, followed by different letters representing significant differences (Tukey’s test; *p* < 0.05).

The linear relationship between the exendin-4 dose and larval mortality was determined by the following equation: *Y* = −0.934 + 0.017*X*, R^2^ = 0.644. A significant positive correlation was observed between the exendin-4 dose and larval mortality, with a correlation coefficient of *r* = 0.831 (*F* = 76.385; *p* < 0.05) (Figure 1; Table 1). The low-lethal (LC_20_) and half-lethal (LC_50_) concentrations of exendin-4 in larvae at 72 h were 5.526 ppm and 55.774 ppm, respectively.

### 3.2. Exendin-4 Arrested Growth and Nutritional Indices

**Figure 2:**
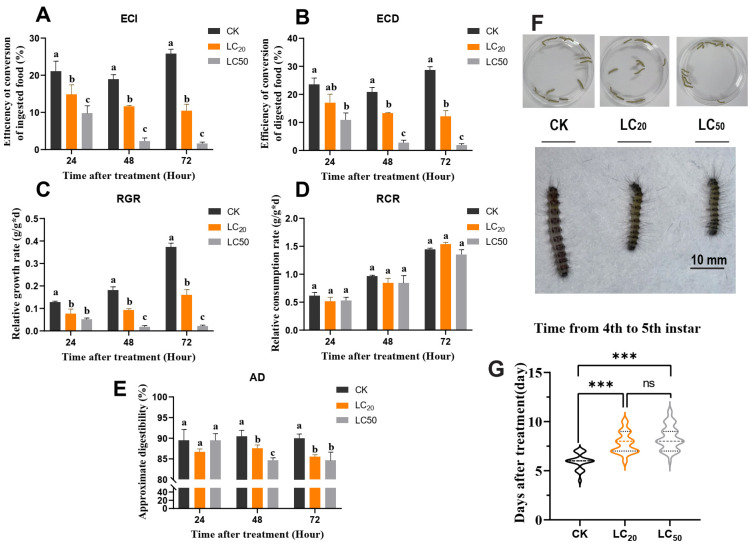
Effects of exendin-4 treatment on growth and nutritional indices of *H. cunea* larvae. (**A**) ECI: efficiency of conversion of ingested food. (**B**) ECD: efficiency of conversion of digested food. (**C**) RGR: relative growth rate. (**D**) RCR: relative consumption rate. (**E**) AD: approximate digestibility. (**F**) The growth under each treatment compared at 72 h. (**G**) The molting time from the 4th to 5th instar of larvae after exendin-4 treatment. Data in (**A**–**E**) are presented as means ± SE of three independent biological replications, followed by different letters representing significant differences (Tukey’s test; *p* < 0.05). Data in (**G**) were analyzed by one-way ANOVA. Asterisks indicate significant differences between each group (*** *p* < 0.001). ns indicates non-significance.

The nutritional indices of fourth-instar *H. cunea* larvae indicated that exendin-4 treatment significantly affected ECI, ECD, RGR, and AD while showing no significant effect on RCR (Figure 2A–E). Exendin-4 (LC_50_ and LC_20_) significantly decreased ECI and ECD in larvae apart from ECD at LC_20_ after 24 h of treatment (Figure 2A,B). There was no impact on RCR, but RGR was significantly decreased beginning at 24 h post-feeding at both concentrations (Figure 2C,D). Meanwhile, AD was significantly suppressed at 48 h and 72 h following exendin-4 (LC_50_ and LC_20_) treatment (Figure 2E). In addition, the inhibition by exendin-4 of larval growth was not only reflected in the retardation of larval body size but also the change in the instar duration. The molting time from the fourth to fifth instar was significantly increased in the LC_20_-treated group (7.824 ± 1.068) and the LC_50_-treated group (8.341 ± 1.217) compared with the CK group (5.955 ± 0.776). Furthermore, the inhibition by exendin-4 of larval growth was evident not only in the delayed development of larval body size but also in the alteration of the instar duration (Figure 2F,G). The duration of molting from the fourth to the fifth instar was significantly prolonged in the LC_20_ group (7.824 ± 1.068 days) and LC_50_ group (8.341 ± 1.217 days) compared with the CK group (5.955 ± 0.776 days). Together, exendin-4 treatment negatively influenced growth and nutritional indices in larvae, with significant inhibitory effects observable even at the lower concentration of LC_20_ (Figure 2G).

### 3.3. Exendin-4 Affected the Activities of Digestive Enzymes

**Figure 3:**
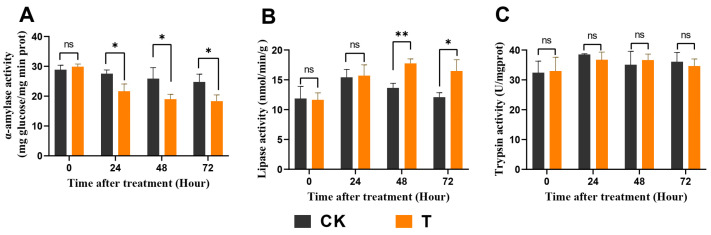
Effects of exendin-4 on digestive enzyme activities in midgut. (**A**) α-amylase activity. (**B**) Lipase activity. (**C**) Trypsin activity. Data were analyzed by one-way ANOVA. Asterisks indicate significant differences between each group (* *p* < 0.05; ** *p* < 0.01). ns indicates non-significance.

The effect of exendin-4 on the digestive function of *H. cunea* larvae revealed significant influences on the activity of larval midgut digestive enzymes. Specifically, there was a significant decrease in the activity of α-amylase, while the activity of lipase showed a significant increase (Figure 3A,B). Following exendin-4 treatment for 24 h, 48 h, and 72 h, the activity of α-amylase decreased by 21.28%, 26.56%, and 25.99%, respectively, compared with the control group (Figure 3A). Conversely, the lipase activity increased by 30.45% and 36.59% after 48 h and 72 h of exendin-4 treatment, respectively (Figure 3B).

### 3.4. Metabolomics Analysis of H. cunea Larvae’s Response to Exendin-4 Treatment

**Figure 4:**
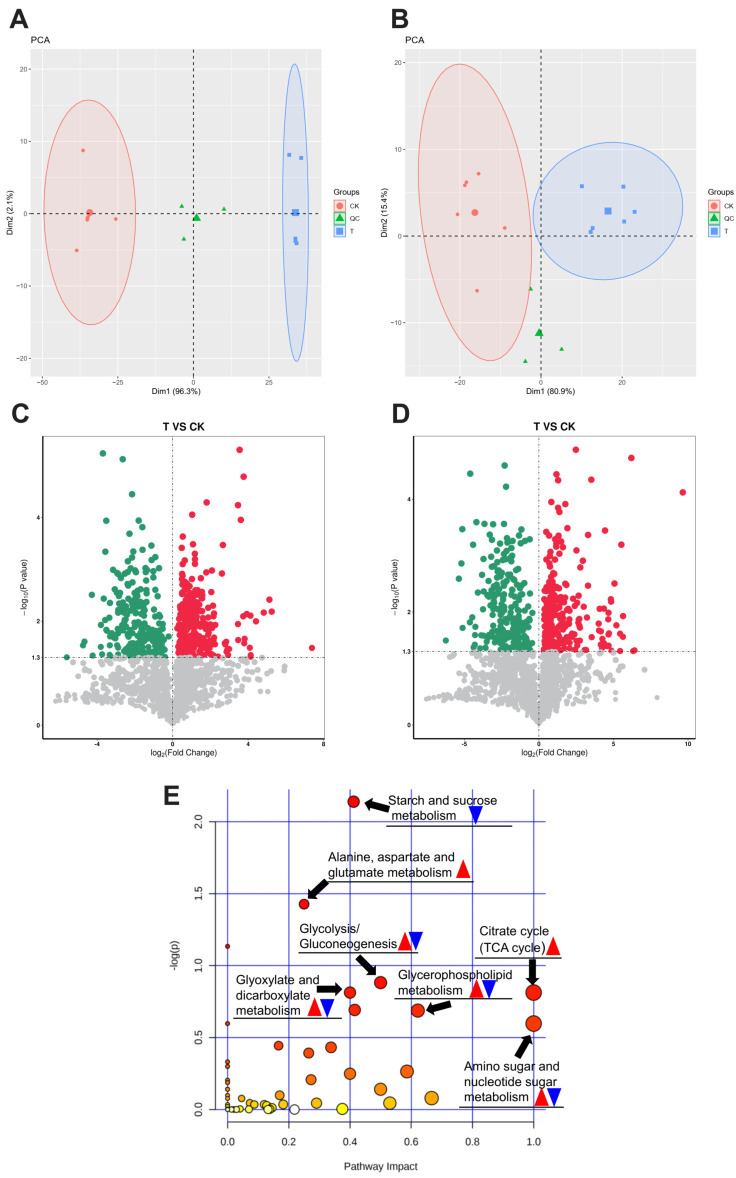
Metabolomics analysis of *H. cunea* larvae with exendin-4 (LC_20_) treatment and CK groups. The PCA plots of each group for the data collected in (**A**) positive-ion mode and (**B**) negative-ion mode, with red, blue, and green representing the CK, T, and QC groups, respectively. Volcano plots displaying the DEMs in the exendin-4-treated group compared with the CK group (**C**,**D**). The dots show the DEMs that were significantly down-regulated (green) or up-regulated (red). (**E**) KEGG pathway enrichment analysis of DEMs between exendin-4 and CK groups. The color’ s depth of the bubbles represents the *p*-value of the enrichment analysis, with red indicating a more significant enrichment, and white or light yellow indicating less significant enrichment. The red and blue triangles represent the up-regulation and down-regulation of differential metabolites in the pathway, respectively.

LC-MS-based metabolomics analysis was utilized to investigate various metabolic pathways in larvae. The results of the PCA clearly separated the CK and T groups, with three quality control (QC) samples forming distinct clusters (Figure 4A,B). Metabolites identified with VIP values of > 1 and *p*-values of < 0.05 (Student’s *t*-test) were considered differentially expressed metabolites (DEMs), resulting in a total of 538 DEMs in the positive-ion mode and 516 DEMs in the negative-ion mode, as illustrated in volcano plots (Figure 4C,D). Subsequent KEGG pathway enrichment analysis was conducted to elucidate the enrichment of DEMs in different metabolic pathways (Figure S3A,B). The highest response was chosen among the positive and negative modes when encountering duplicate metabolites. The DEMs were primarily enriched in pathways related to energy substance metabolism, including the citrate cycle (TCA cycle), amino sugar and nucleotide sugar metabolism, starch and sucrose metabolism, glycolysis/gluconeogenesis, glycerophospholipid metabolism, alanine, aspartate and glutamate metabolism, as well as glyoxylate and dicarboxylate metabolism (Figure 4E). The regulation of DEMs within the aforementioned metabolic pathways is shown in Table 2 and Figure S4A–G.

### 3.5. Exendin-4 Affected Carbohydrate Levels

**Figure 5:**
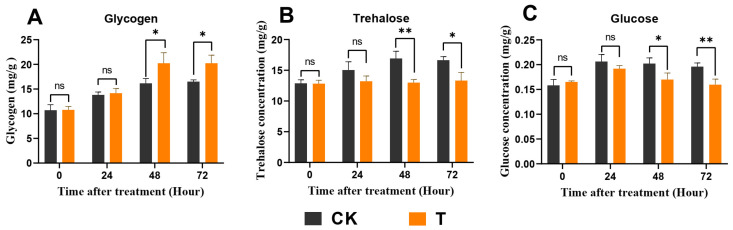
Effects of exendin-4 treatment on key carbohydrate metabolites. (**A**) Glycogen in fat body. (**B**) Trehalose in hemolymph. (**C**) Glucose in hemolymph. Data were analyzed by one-way ANOVA. Asterisks indicate significant differences between each group (* *p* < 0.05; ** *p* < 0.01). ns indicates non-significance.

The impact of exendin-4 on carbohydrates was primarily evident after 48 h and 72 h of treatment. There was a notable increase in the glycogen content in the larval fat body, while the levels of glucose and trehalose in the larval hemolymph significantly decreased (Figure 5A–C).

**Figure 6:**
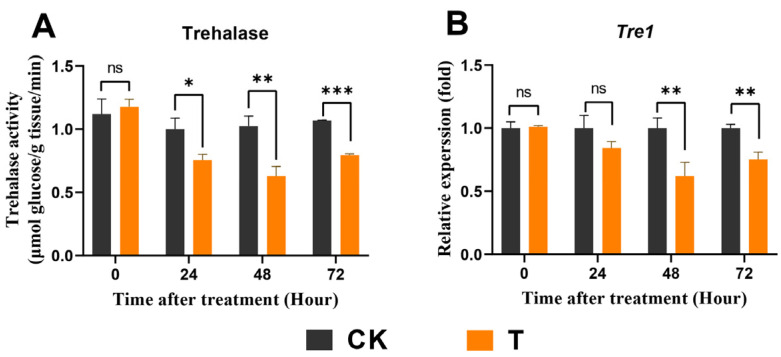
Effects of exendin-4 on the activities of trehalase and *Tre1* gene expression. (**A**) Trehalase activity. (**B**) Relative expression of *Tre1* gene. Data were analyzed by one-way ANOVA. Asterisks indicate significant differences between each group (* *p* < 0.05; ** *p* < 0.01; *** *p* < 0.001). ns indicates non-significance.

Moreover, to further analyze the effects of exendin-4 on trehalose and glucose homeostasis in larval blood sugar, the activities of trehalase and the relative expression of *Tret1* were determined [57]. Compared with CK, the activity of trehalase significantly decreased after 24 h of treatment (Figure 6A), with a concurrent down-regulation in the expression of *HcTret1* after 48 h of treatment (Figure 6A,B). The regulation of enzyme activity, gene expression, and metabolite levels were regulated by different mechanisms but also showed synchronous trends to some extent.

### 3.6. Exendin-4 Promoted Glycolysis

**Figure 7:**
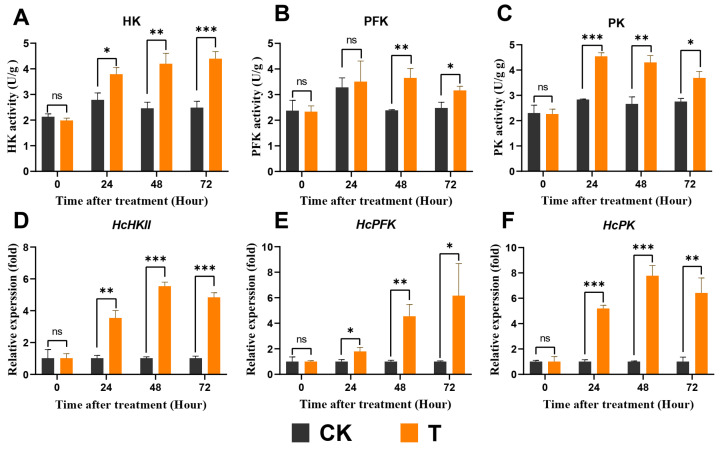
Effects of exendin-4 on the activities of glycolytic rate-limiting enzymes and key genes’ expression. (**A**) HK activity. (**B**) PFK activity. (**C**) PK activity. (**D**) Relative expression of *HcHKII* gene. (**E**) Relative expression of *HcPFK* gene. (**F**) Relative expression of *HcPK* gene. Data were analyzed by one-way ANOVA. Asterisks indicate significant differences between each group (* *p* < 0.05; ** *p* < 0.01; *** *p* < 0.001). ns indicates non-significance.

The activities of three rate-limiting enzymes (HK, PFK, and PK) involved in glycolysis in larvae showed significant enhancement with an increasing exendin-4 treatment duration compared with CK (Figure 7A–C). Additionally, the relative expressions of *HcHKII*, *HcPFK*, and *HcPK* were measured. In comparison with CK, the mRNA expressions of *HcHKII*, *HcPFK*, and *HcPK* were significantly up-regulated after 24 h of treatment (Figure 7D–F). The regulation of genes and enzyme activities operates at different levels, which may hinder the synchronous expression of outcomes. In general, these data suggest that exendin-4 resulted in an increased glycolysis flux in *H. cunea* larvae.

### 3.7. Exendin-4 Affected PA and ATP Levels

**Figure 8:**
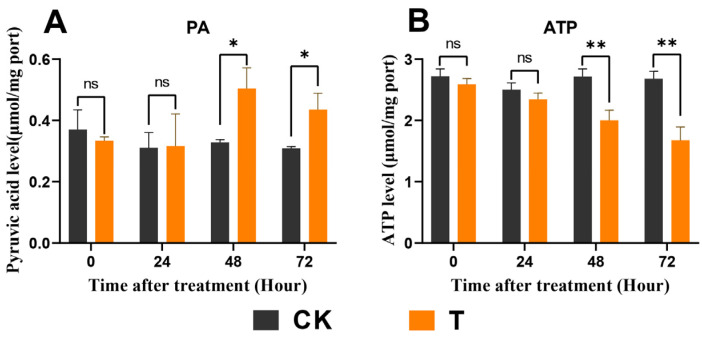
Effects of exendin-4 on PA and ATP contents. (**A**) PA content. (**B**) ATP content. Data were analyzed by one-way ANOVA. Asterisks indicate significant differences between each group (* *p* < 0.05; ** *p* < 0.01). ns indicates non-significance.

ATP and PA are products of glycolysis, and their levels were measured. Exendin-4 treatment had a significant effect on the PA content in larvae after 48 h (Figure 8A). Meanwhile, the results for ATP levels showed significant differences after 48 h of treatment (Figure 8B).

### 3.8. Exendin-4 Regulated Expressions of Key Genes Related to AMPK and ILP Pathways

**Figure 9:**
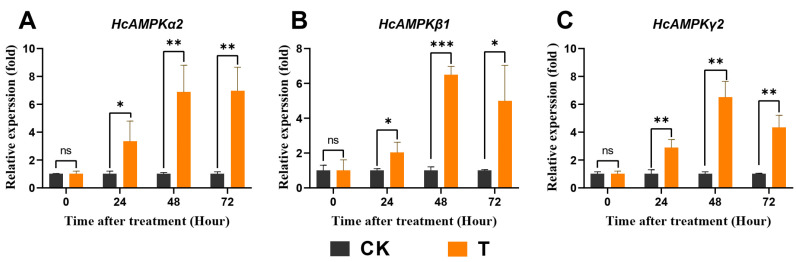
Effects of exendin-4 treatment on the relative expressions of three key genes involved in AMPK pathways. (**A**) Relative expression of *HcAMPKα2*. (**B**) Relative expression of *HcAMPKβ1*. (**C**) Relative expression of *HcAMPKγ2*. Data were analyzed by one-way ANOVA. Asterisks indicate significant differences between each group (* *p* < 0.05; ** *p* < 0.01; *** *p* < 0.001). ns indicates non-significance.

**Figure 10:**
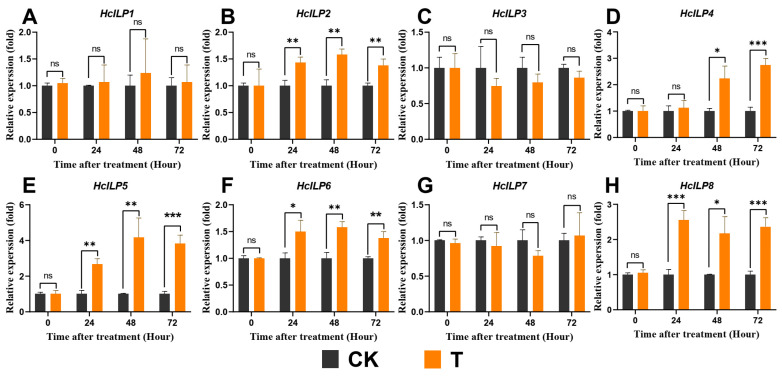
Effects of exendin-4 treatment on insulin-like peptide expression. (**A**–**H**) show the relative expressions of key genes involved in insulin-like peptide expression. Data were analyzed by one-way ANOVA. Asterisks indicate significant differences between each group (* *p* < 0.05; ** *p* < 0.01; *** *p* < 0.001). ns indicates non-significance.

To further estimate the regulatory effects of exendin-4 on the energy homeostasis of *H. cunea* larvae, the relative expressions of key genes involved in the AMPK and ILP pathways were determined. Exendin-4 significantly upregulated the mRNA expressions of *HcAMPKα2*, *β1*, and *γ2* after 24 h of treatment (Figure 9A–C). Notably, the increases in the three genes corresponded with the trend of ATP level decrease (Figure 8B and Figure 9A–C). The effects on *HcILP* transcription were observed for *HcILP2* (24 h to 72 h), *HcILP4* (48 h to 72 h), *HcILP5* (24 h to 72 h), *HcILP6* (24 h to 72 h), and *HcILP8* (24 h to 72 h), which were significantly higher (1.38–4.18-fold) compared with CK (Figure 10A–H). Although the changes in the ILPs’ expressions were not substantial in terms of fold change compared with AMPK, such variations could still exert significant effects on physiological functions.

## 4. Discussion

Insects adapt to stress conditions that hinder their growth and development by actively adjusting their nutritional indices to optimize digestion and food utilization [58]. Extensive research indicates that insect growth regulators (IGRs) suppress larval feeding and digestive efficiency and even resist feeding after use [47,49]. Similarly, in this study, exendin-4 treatment significantly down-regulated the growth and nutritional indices of *H. cunea* larvae, suggesting that exendin-4 inhibited larval feeding and decreased the efficiency of nutrient conversion into body mass, ultimately affecting weight gain (Figure 2A–E). Notably, RCR was not significantly affected, indicating that exendin-4 treatment did not directly interfere with larval feeding behavior (Figure 2D). Further analysis revealed a significant decrease in α-amylase activity and an increase in lipase activity in the larval midgut (Figure 3A,B). Previous studies have shown that GLP-1R agonists impact digestive enzyme levels by regulating pancreatic acinar cell proliferation in mammals [59]. Therefore, exendin-4 may have a potential target site in the larval midgut, influencing food digestion strategies by regulating α-amylase and lipase activities. Moreover, in addition to arresting larval growth, exendin-4 treatment also significantly prolonged the duration of the fourth instar (Figure 2F,G). Based on these findings, exendin-4 shows promising potential to be an IGR.

Changes in biological nutritional absorption strategies are typically accompanied by significant alterations in energy substances and metabolic profiles within the organism [60]. Studies have shown that GLP-1R agonists can regulate the metabolism and utilization of energy substrates in mammals [60,61]. In the present study, numerous DEM responses to exendin-4 treatment were identified in *H. cunea* larvae (Figure 4A–D). According to the results of the KEGG analysis, the DEMs were mainly enriched in seven metabolic pathways (Figure S4 and Figure 4E). Most of the metabolites involved were glycolytic substrates and their precursors, or intermediate products of the TCA cycle (Table 2 and Figure S4A–G). Thus, it can be confirmed that exendin-4 significantly influences the energy metabolism of the larvae.

In mammals, the impact of GLP-1R agonists on energy metabolism primarily manifests through their regulation of blood glucose levels mediated by insulin [38]. By promoting the uptake and utilization of glucose by cells, these agonists lower blood glucose and stimulate glycogen synthesis [32]. Similar to insulin’s action, insect insulin-like peptides induce the conversion of glucose into glycogen following its uptake by cells [62]. Interestingly, in this study, exendin-4 treatment demonstrated a similar regulatory effect on blood sugar homeostasis in *H. cunea* larvae. The glucose and trehalose levels in the larval hemolymph significantly decreased, while the glycogen content in the fat body notably increased (Figure 5A–C). The reduction in trehalose, which functions as blood sugar in insects, was typically due to the increased activity of trehalase driven by downstream energy demands (Figure 6A,B) [63]. The decrease in glucose levels and increase in the glycogen content may be due to the activation of ILPs’ secretion by exendin-4 treatment (Figure 10A–H). Based on these results, exendin-4 directly regulated sugar metabolism in *H. cunea* larvae; however, further research is needed to clarify its receptor and function. Additionally, the increased rate of glycolysis elevated the demand for substrate glucose, also contributing to the reduced glucose levels in the hemolymph (Figure 7A–F).

Glycolysis is the primary dissimilatory pathway responsible for breaking down glucose to extract energy in animal cells [26]. In insects, the final product of glycolysis, PA, is transported into the mitochondria, where it facilitates ATP production through the TCA cycle, thus playing a crucial role in nutrient metabolism at physiological levels [31]. It has been reported that the stress of exogenous substances can regulate pathways involved in glycolysis and affect related enzyme activities in insects [29,64,65]. Meanwhile, GLP-1R activation was found to augment glycolytic levels in mammals [36]. In this study, exendin-4 enhanced the activities as well as mRNA expressions of glycolytic rate-limiting enzymes (HK, PFK, and PK) (Figure 7A–F) and, finally, produced more PA (Figure 8A), which indicated that the glycolytic flux increased in *H. cunea* larvae. Furthermore, the activities of PFK and PK are modulated by allosteric regulation involving catalytic products, as well as by intracellular ATP/ADP levels, insulin-related signaling pathways, and the AMPK signaling pathway, all of which were significantly influenced by exendin-4 treatment in this study (Figure 8A,B, Figure 9A–C, and Figure 10A–H) [24,66,67].

However, further results revealed a significant reduction in ATP levels in the larvae after 48 h of exendin-4 treatment (Figure 8B). Several explanations could account for this observation. Firstly, the TCA cycle, an essential pathway for ATP production, showed increased levels of various intermediates in the larvae following exendin-4 treatment (Table 2 and Figure S4B). For example, oxaloacetate, succinate, and citrate play roles in the production and replenishment of ATP for insects [68]. Secondly, as an exogenous regulator, exendin-4 may activate detoxification metabolism in larvae, which is energetically costly [28]. ATP is utilized to provide more energy for detoxification enzyme cells and for ATP-binding cassette (ABC) transporters to expel metabolites [69]. Thirdly, GLP-1R agonists are positively coupled to adenylate cyclase via Gαs-containing G proteins, which catalyze the conversion of ATP into cyclic adenosine monophosphate (cAMP) [70]. Exendin-4 may also regulate ATP levels through this signaling pathway in larvae.

As an energy sensor, AMPK is activated by variations in intracellular ATP levels, leading to arresting ATP-consuming biosynthetic pathways, inhibiting multiple anabolic pathways, and the negative regulation of larval growth and nutrient uptake [47,71]. In the present study, a tight and negative correlation between the mRNA expressions of *HcAMPK α2*, *β1*, and *γ2* and ATP levels was found (Figure 9A–C). AMPK activation also provided a basis for exendin-4 suppressing the growth and nutritional indices of *H. cunea* larvae. In addition, it has been reported that GLP-1R agonists stimulate the phosphorylation of the AMPK α-subunit at Thr172, thereby activating AMPK [72]. Therefore, exendin-4 may directly activate AMPK in *H. cunea* larvae through this mechanism. Concurrently, AMPK is involved in insulin signaling, thereby controlling glucose uptake and insulin sensitivity [67]. Although the findings of this study reveal the influence of exendin-4 on AMPK and ILPs in *H. cunea* larvae (Figure 9A–C and Figure 10A–H), further exploration of the synergistic interactions between related signaling pathways is warranted.

## 5. Conclusions

In conclusion, the present study demonstrated the regulatory effects of exendin-4 on larval growth and sugar metabolism in *H. cunea* larvae, combined with metabolomics analysis to elucidate the multiple metabolic pathways involved in glycolysis and the TCA cycle. The findings indicate that exendin-4 up-regulated ILPs, regulated sugar homeostasis, promoted glycolysis, reduced ATP levels, activated AMPK, and, ultimately, resulted in the arrest of growth and nutritional indices in *H. cunea* larvae. According to the results of this study, we propose a schematic description of the mechanism of exendin-4 in *H. cunea* larvae, as presented in the Graphic Abstract. However, as a class of GLP-1R agonists, the action mechanism of exendin-4 has not been fully revealed, especially the identification and function of its receptor. From the perspective of energy metabolism, further exploration of the action mechanism of exendin-4 is warranted in insects. Furthermore, it is noteworthy that exendin-4 treatment did not directly interfere with larval feeding behavior but instead induced growth arrest and, at higher concentrations, lethality. These characteristics suggest that exendin-4 could potentially be used to mitigate the growth and development of phytophagous pests, thereby reducing their damage to plants. Therefore, future research should explore the application potential of exendin-4 in pest management, providing new strategies for plant protection.

## Figures and Tables

**Table 1:** Test of toxicity of exendin-4 to *H. cunea* larvae.

Exposure Time (h)	LC_20_ (ppm)	95% Confidence Interval	LC_50_ (ppm)	95% Confidence Interval	R^2^	Linear Equation
72	5.526	1.969–12.084	55.744	48.398–64.686	0.644	Y = −0.934 + 0.017X

**Table 2:** Significantly enriched metabolic pathways and DEMs.

Metabolic Pathways	Differentially Expressed Metabolites (DEMs)	
	Up	Down
Citrate cycle (TCA cycle)	Dihydrolipoamide-E Citrate Fumaric acid Pyruvate Oxaloacetate Succinate Thiamin diphosphate Phosphoenolpyruvate 2-oxoglutarate	
Amino sugar and nucleotide sugar metabolism	N-acetylchitosamine N-acetyl-D-mannosamine α-D-glucose GDP-L-fucose	D-fructose 6-phosphate D-fructose D-mannose ADP-glucose Glucose 1-phosphate Glucose 6-phosphate
Starch and sucrose metabolism		Trehalose Sucrose D-glucose 1-phosphate D-fructose 6-phosphate D-fructose ADP-glucose
Glycolysis/gluconeogenesis	Pyruvate Oxaloacetate α-D-glucose Dihydrolipoamide-E Thiamin diphosphate Phosphoenolpyruvate	D-glucose 6-phosphate D-glucose 1-phosphate D-fructose 6-phosphate
Glycerophospholipid metabolism	Choline Acetylcholine Ethanolamine	Phosphatidylethanolamine Phosphatidylcholine O-phosphocholine
Alanine, aspartate, and glutamate metabolism	Pyruvate Fumarate Succinate Oxaloacetate 2-oxoglutarate Citrate	
Glyoxylate and dicarboxylate metabolism	Formate Oxalate	Glycolaldehyde Hydroxypyruvate

## Data Availability

The raw data supporting the conclusions of this article will be made available by the authors on request.

## Supplementary Materials

The following supporting information can be downloaded at https://www.mdpi.com/article/10.3390/insects15070503/s1. Figure S1. The chemical structure of exendin-4 (CAS no.: 141758-74-9). Figure S2. Effects of 0.01 mM PBS treatment on *H. cunea* larvae. (A) A–H show the relative expressions of key genes involved in insulin-like peptide expression; (B) A–B show trehalose and glucose levels in hemolymph. Data were analyzed by one-way ANOVA. Figure S3. KEGG pathway enrichment analysis of DEMs. (A) Positive mode; (B) negative mode. Figure S4. Visualization of KEGG pathway. (A) Glycolysis/gluconeogenesis; (B) TCA cycle; (C) glyoxylate and dicarboxylate metabolism; (D) starch and sucrose metabolism; (E) amino sugar and nucleotide sugar metabolism; (F) glycerophospholipid metabolism; and (G) glyoxylate and dicarboxylate metabolism. Table S1. Information on primer sequences used in this study.
