# Supplementary figures and images for: Exendin-4 Caused Growth Arrest by Regulating Sugar Metabolism in Hyphantria cunea (Lepidoptera: Erebidae) Larvae
Source: Insects. 2024 Jul 5;15(7):503. doi: 10.3390/insects15070503 (PMC11276936; doi:10.3390/insects15070503)

# Statistics of Pathway Enrichment (Postive mode)

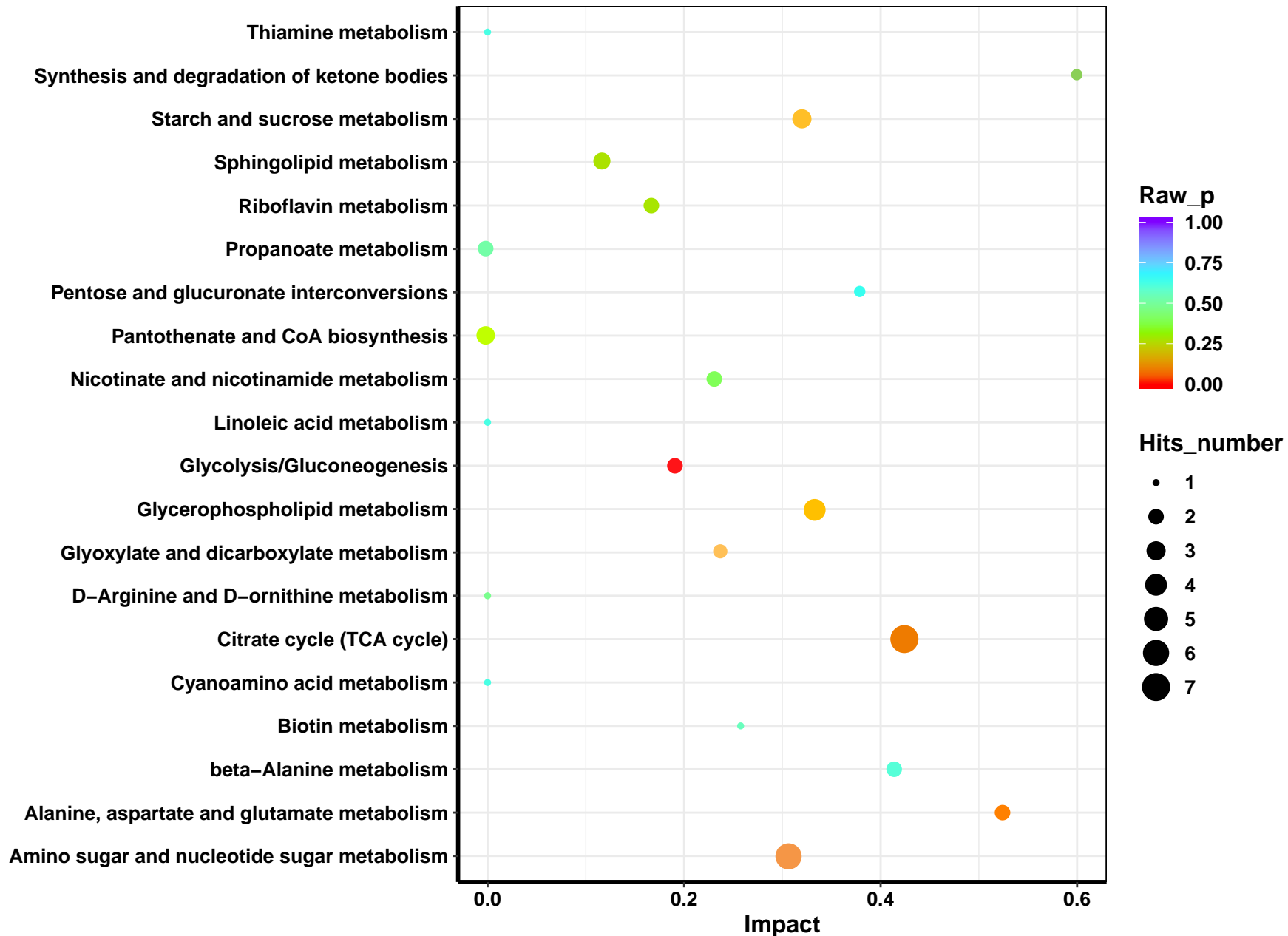

Supplement: Supplementary file 1 [file insects-15-00503-s001.zip › Figure. S3/A.pdf]

# Statistics of Pathway Enrichment (Negative mode)

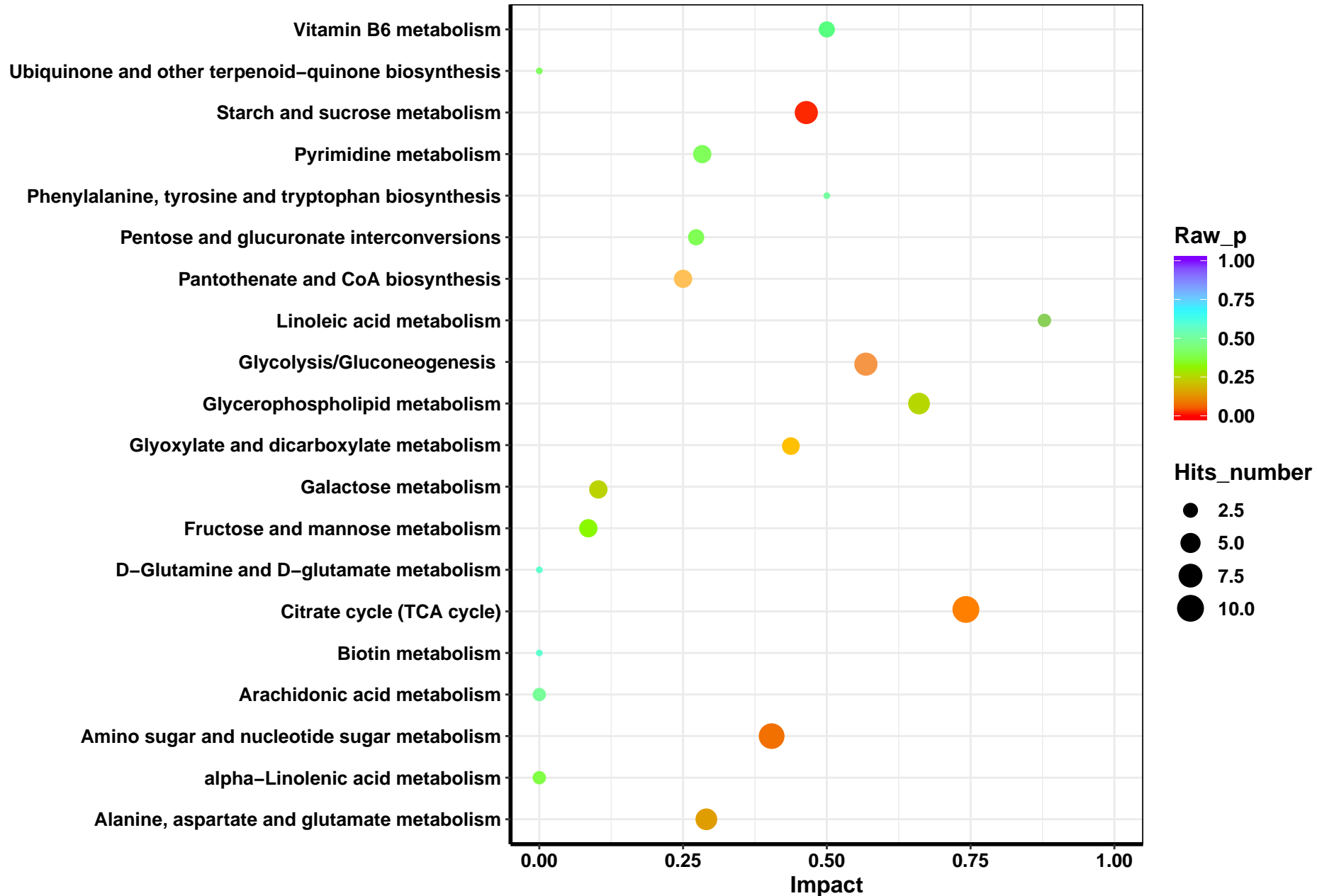

Supplement: Supplementary file 1 [file insects-15-00503-s001.zip › Figure. S3/B.pdf]

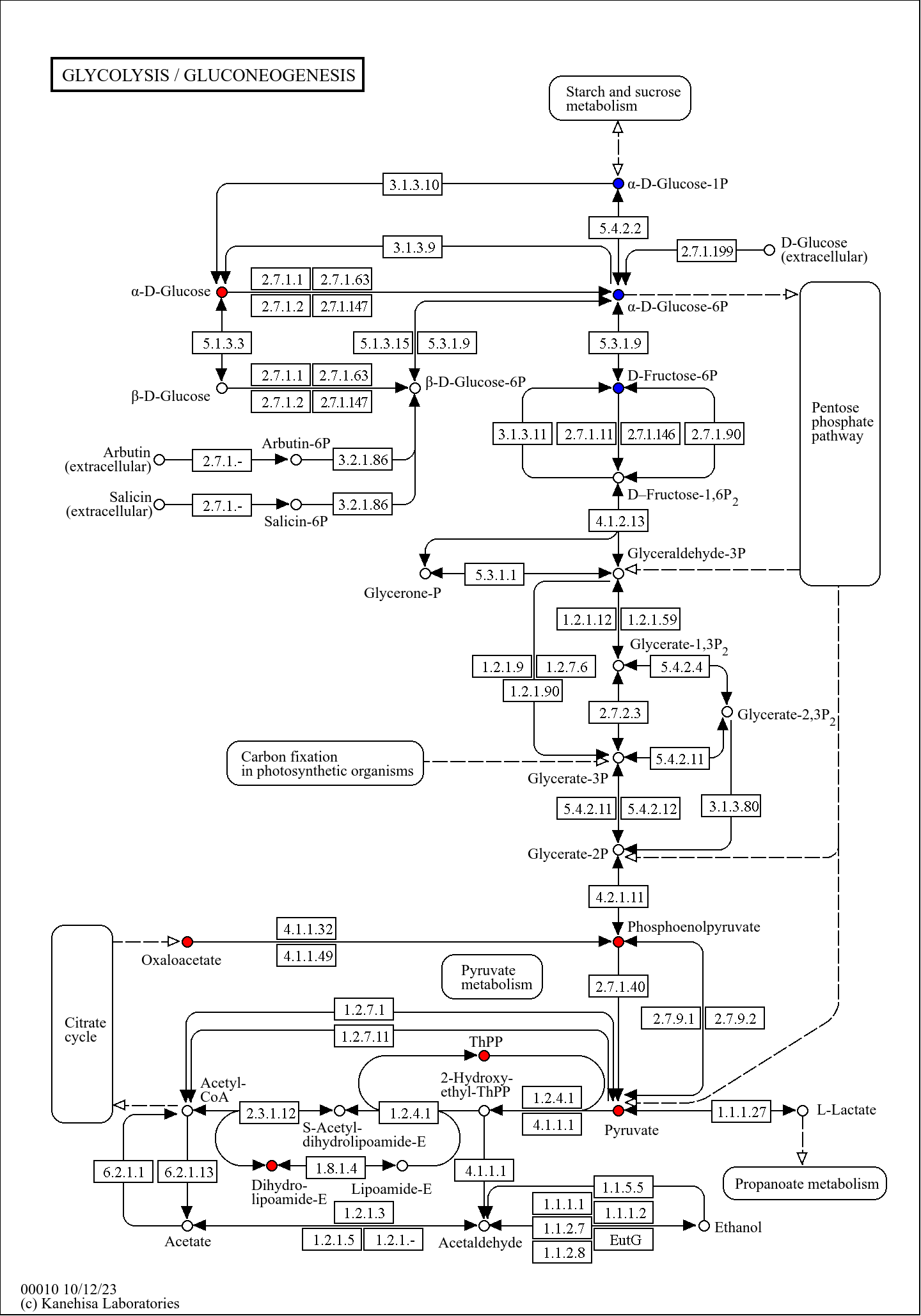

Supplement: Supplementary file 1 [file insects-15-00503-s001.zip › Figure. S4/A. map00010 Glycolysis Gluconeogenesis.png]

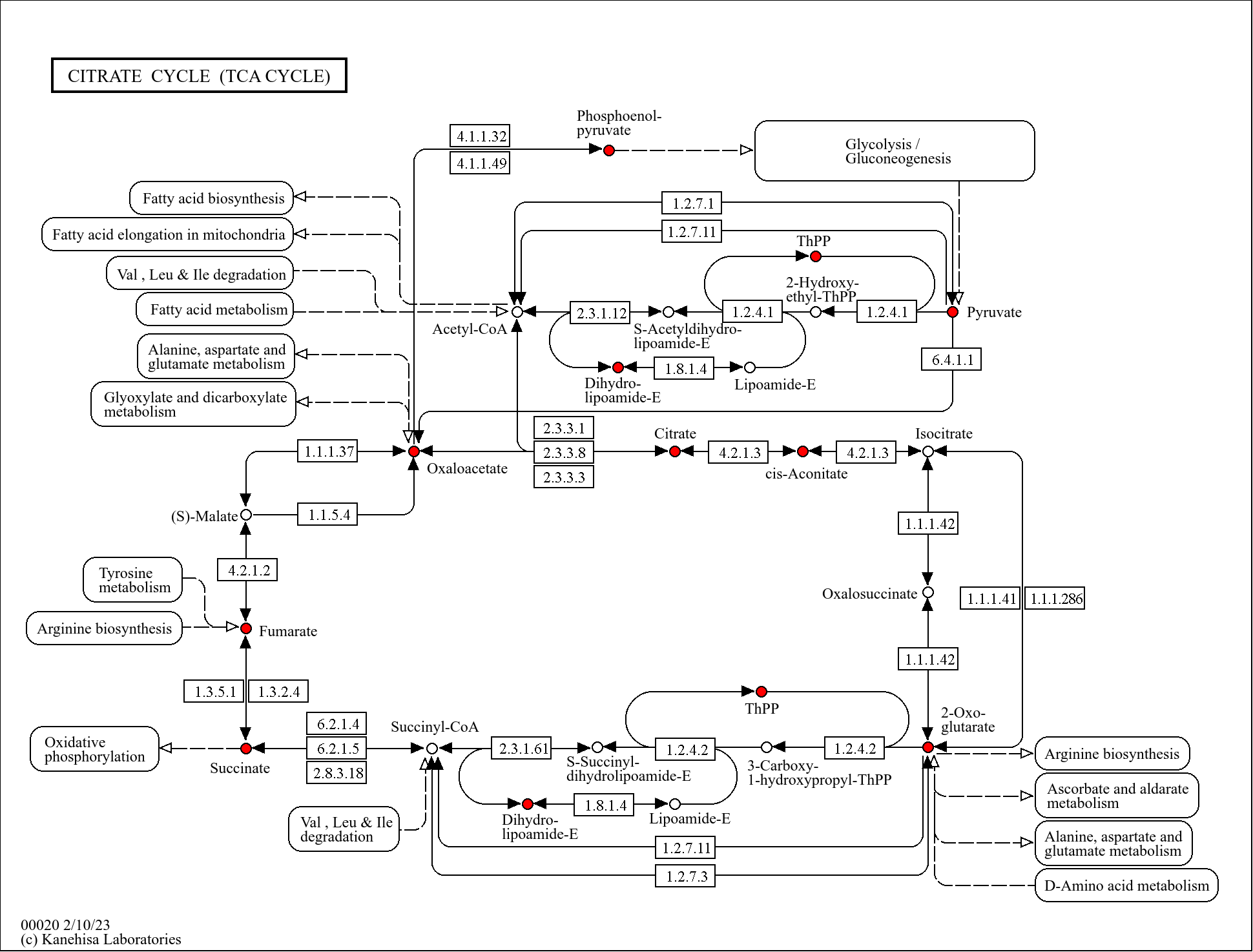

Supplement: Supplementary file 1 [file insects-15-00503-s001.zip › Figure. S4/B. map00020 Citrate cycle (TCA cycle).png]

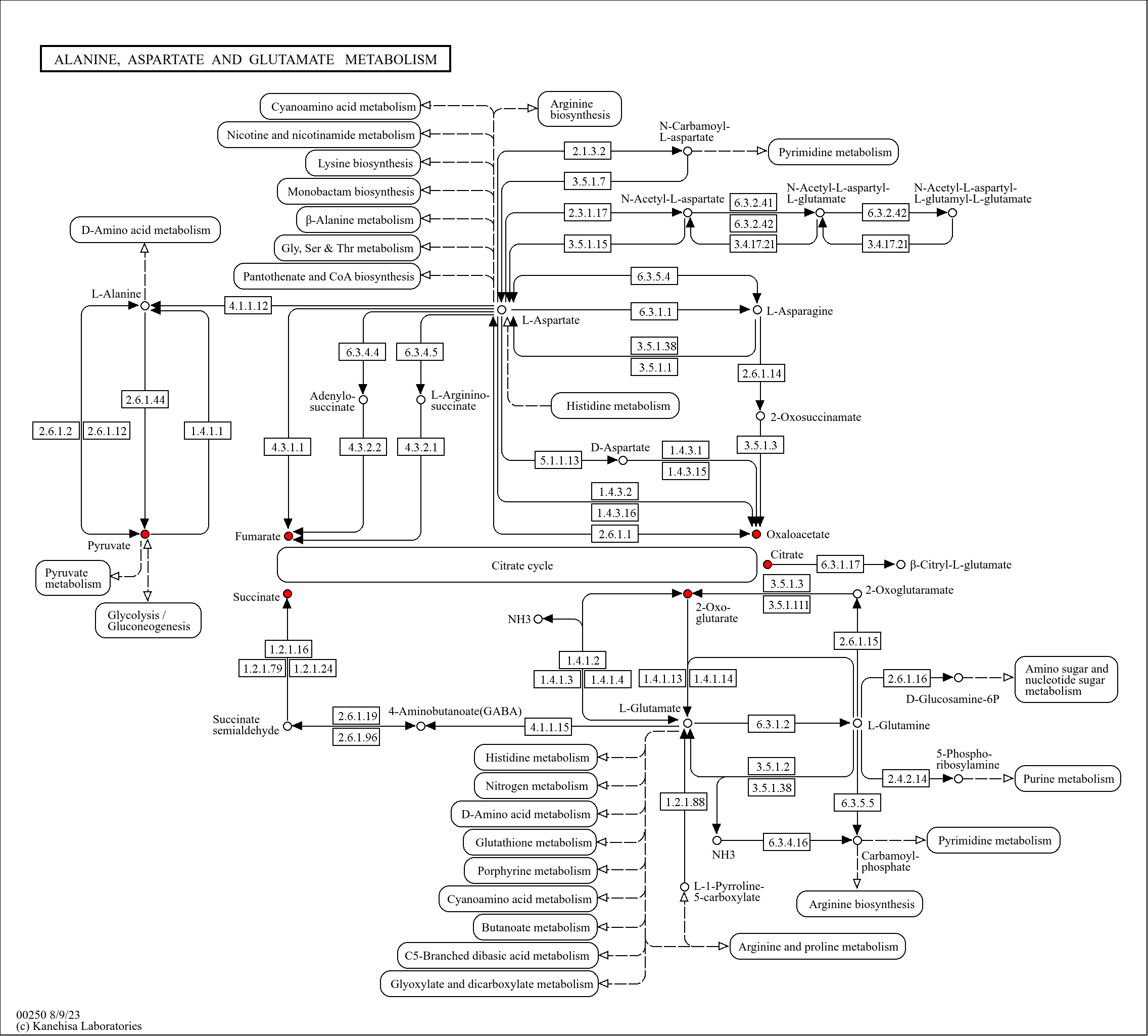

Supplement: Supplementary file 1 [file insects-15-00503-s001.zip › Figure. S4/C. map00250 Glyoxylate and dicarboxylate metabolism.png]

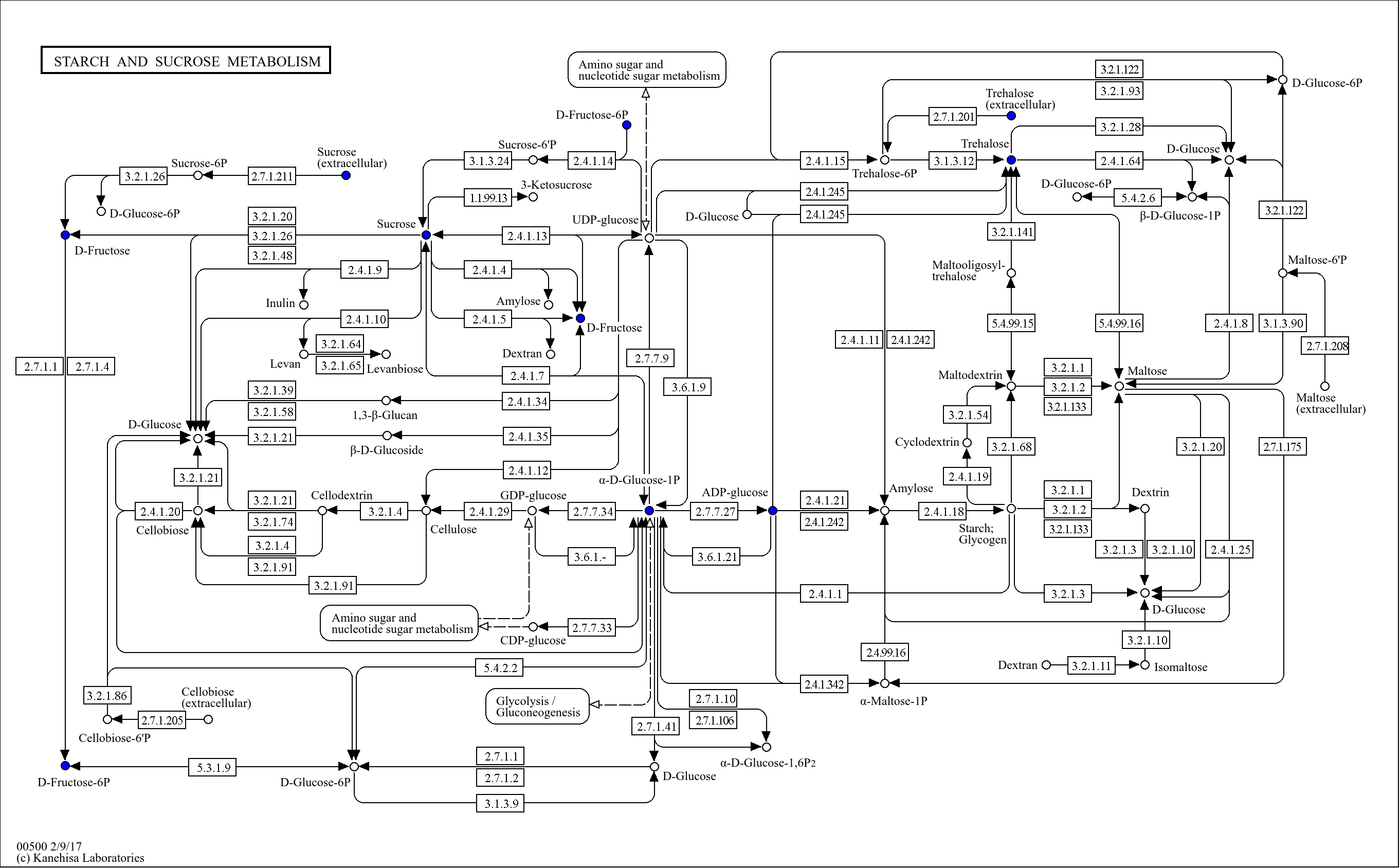

Supplement: Supplementary file 1 [file insects-15-00503-s001.zip › Figure. S4/D. map00500 Starch and sucrose metabolism.png]

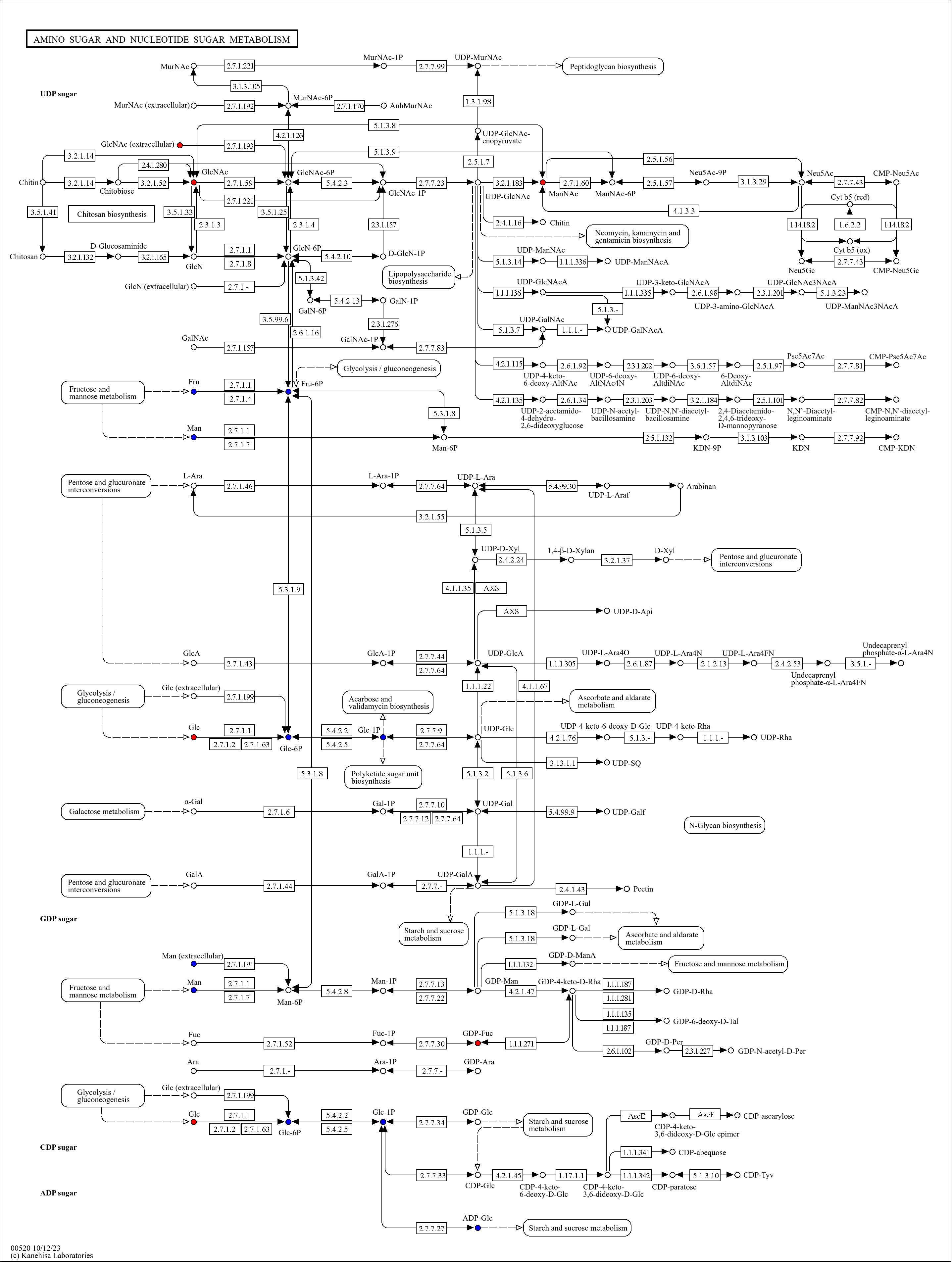

Supplement: Supplementary file 1 [file insects-15-00503-s001.zip › Figure. S4/E. map00520 Amino sugar and nucleotide sugar metabolism.png]

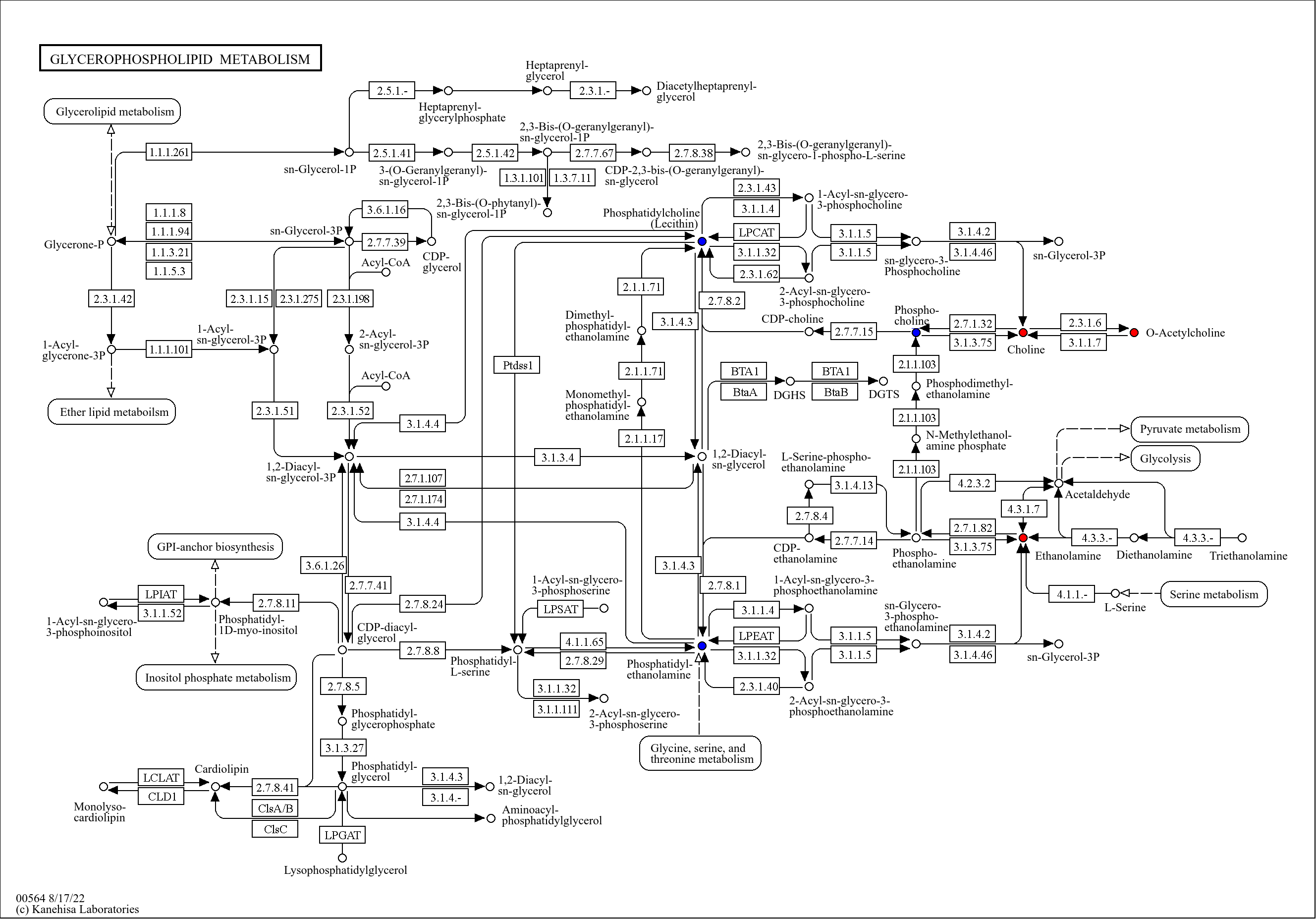

Supplement: Supplementary file 1 [file insects-15-00503-s001.zip › Figure. S4/F. map00564 Glycerophospholipid metabolism.png]

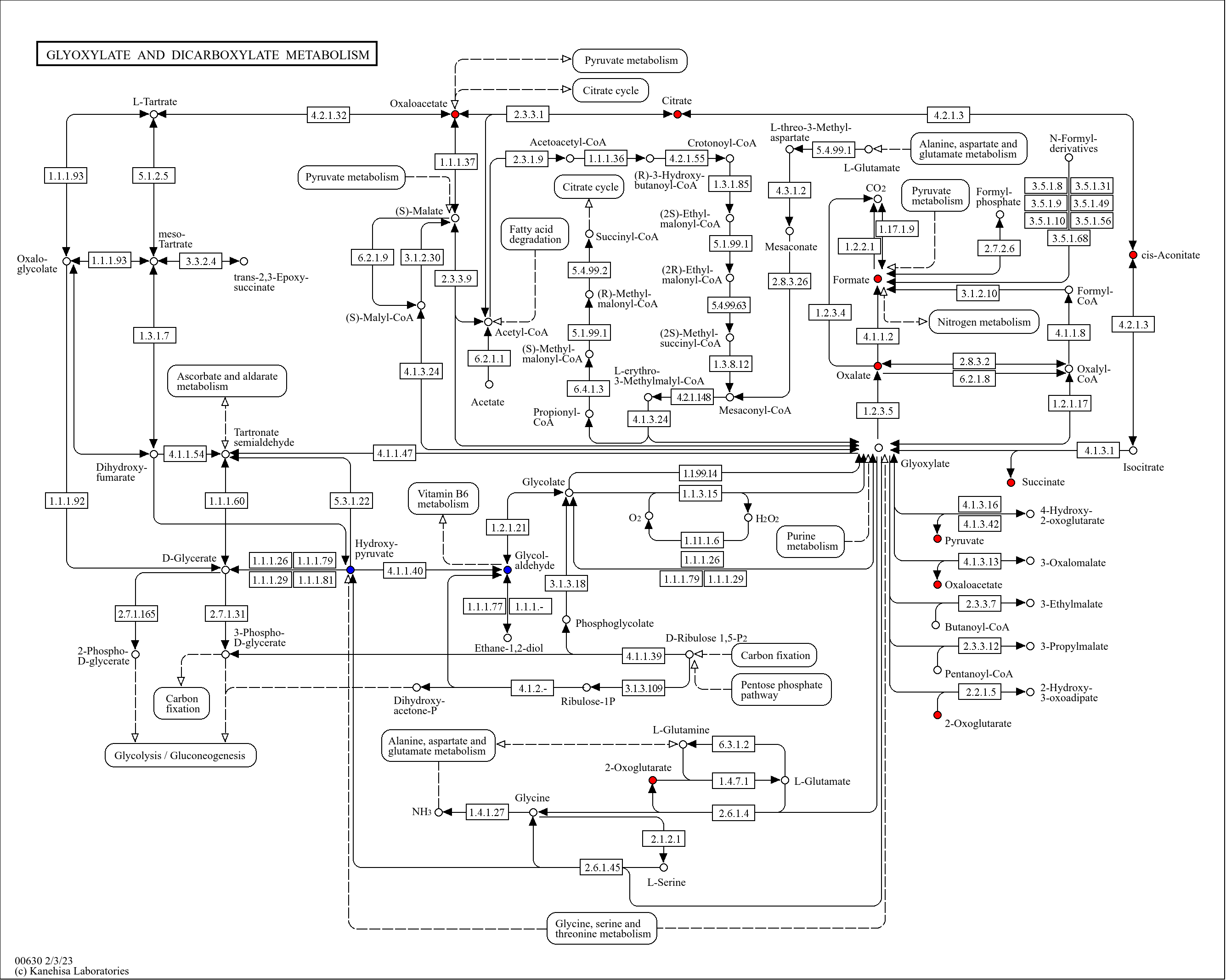

Supplement: Supplementary file 1 [file insects-15-00503-s001.zip › Figure. S4/G. map00630 Glyoxylate and dicarboxylate metabolism.png]

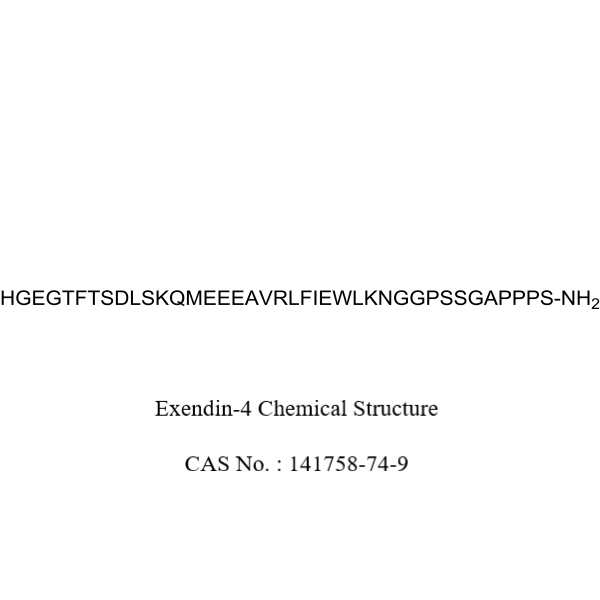

Supplement: Supplementary file 1 [file insects-15-00503-s001.zip › Figure. S1/S1.png]

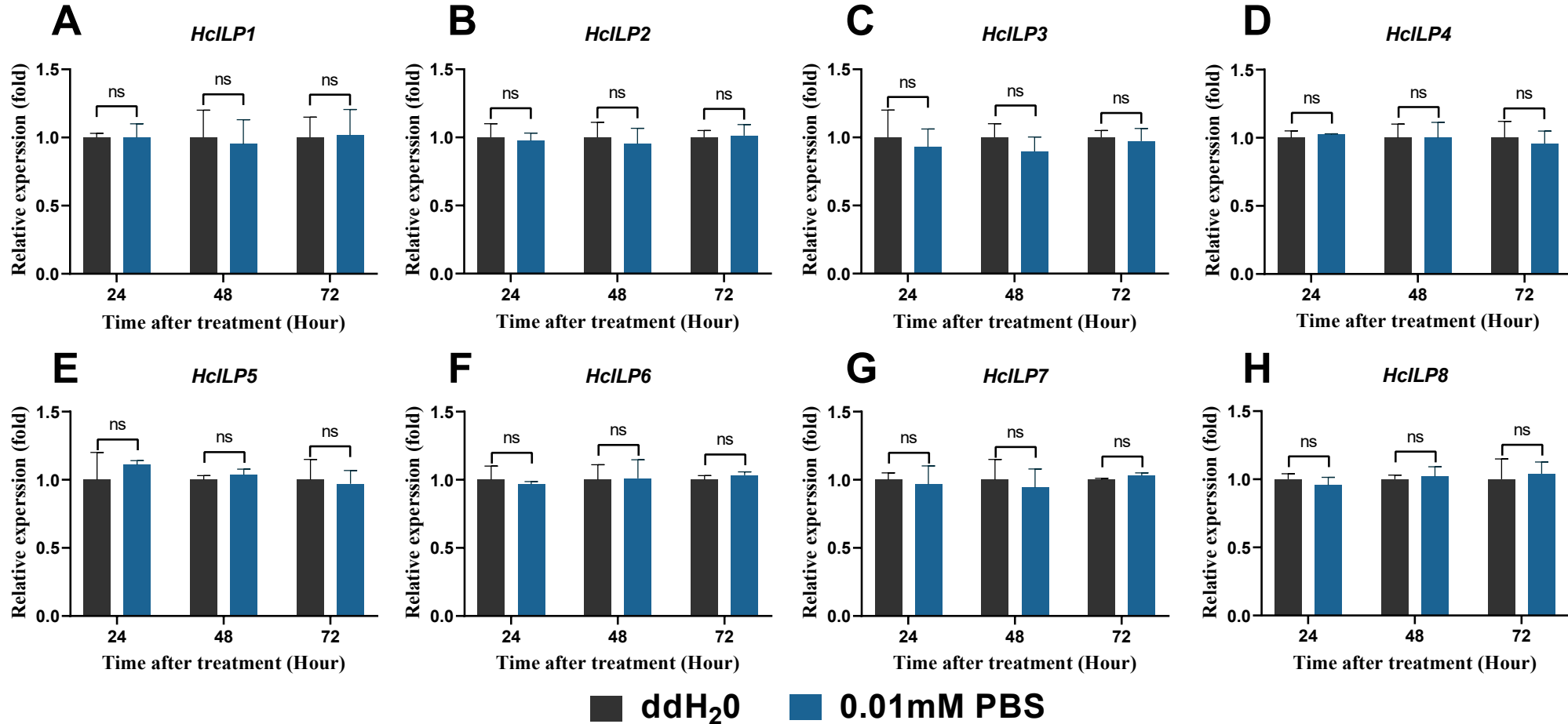

Supplement: Supplementary file 1 [file insects-15-00503-s001.zip › Figure. S2/A.pdf]

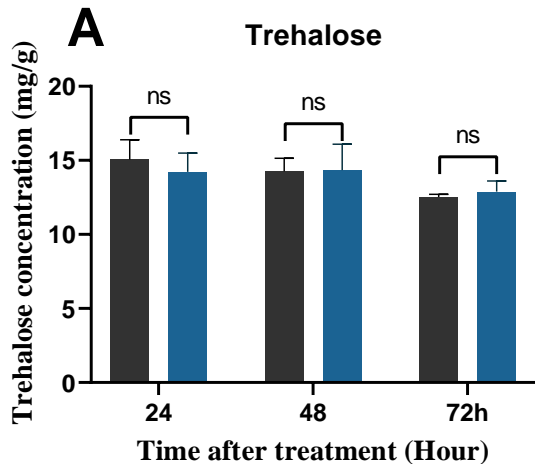

■ ddH<sub>2</sub>O

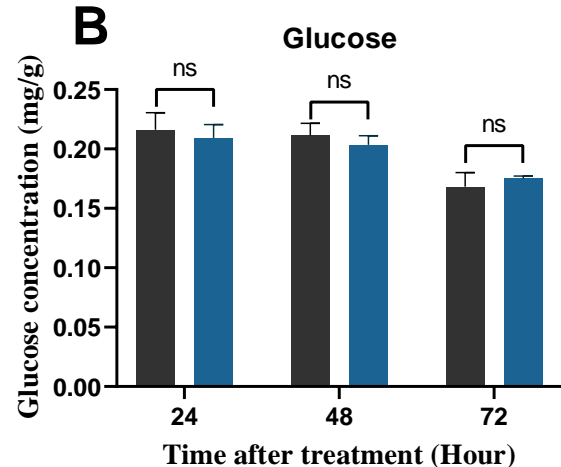

■ 0.01mM PBS

Supplement: Supplementary file 1 [file insects-15-00503-s001.zip › Figure. S2/B.pdf]
